# Metal halide perovskite toxicity effects on *Arabidopsis thaliana* plants are caused by iodide ions

**DOI:** 10.1016/j.isci.2021.103583

**Published:** 2021-12-09

**Authors:** Eline M. Hutter, Reiny Sangster, Christa Testerink, Bruno Ehrler, Charlotte M.M. Gommers

**Affiliations:** 1 Center for Nanophotonics, AMOLF, 1098 XG Amsterdam, the Netherlands; 2 Department of Chemistry, Utrecht University, 3584 CB Utrecht, the Netherlands; 3 Laboratory of Plant Physiology, Wageningen University & Research, 6708 PB Wageningen, the Netherlands

**Keywords:** Inorganic materials, Environmental science, Environmental chemistry, Plant biology, Plants

## Abstract

Highly efficient solar cells containing lead halide perovskites are expected to revolutionize sustainable energy production in the coming years. Perovskites are generally assumed to be toxic because of the lead (Pb), but experimental evidence to support this prediction is scarce. We tested the toxicity of the perovskite MAPbI_3_ (MA = CH_3_NH_3_) and several precursors in *Arabidopsis thaliana* plants. Both MAPbI_3_ and the precursor MAI hamper plant growth at concentrations above 5 μM. Lead-based precursors without iodide are only toxic above 500 μM. Iodine accumulation in *Arabidopsis* correlates with growth inhibition at much lower concentrations than lead. This reveals that perovskite toxicity at low concentrations is caused by iodide ions specifically, instead of lead. We calculate that toxicity thresholds for iodide, but not lead, are likely to be reached in soils upon perovskite leakage. This work stresses the importance to further understand and predict harmful effects of iodide-containing perovskites in the environment.

## Highlights

• Natural *Trichodesmium* colonies collect and maintain dust within their colony core
• Using synthetic particles we tested if colonies select the particles they collect
• Colonies selectively collect and retain nutrient-rich over nutrient-free particles
• Selective collection of particles optimize their nutrient-acquisition from dust

## Graphical abstract

**Figure:**
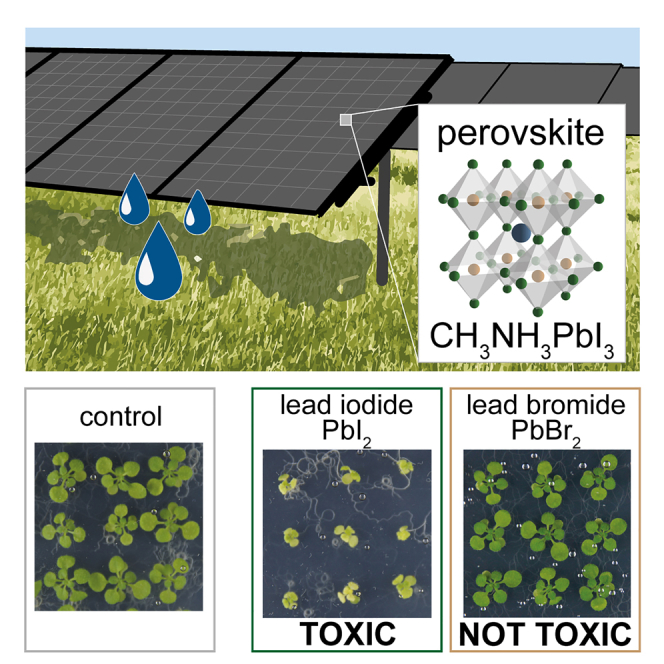

## Introduction

**Figure 1:**
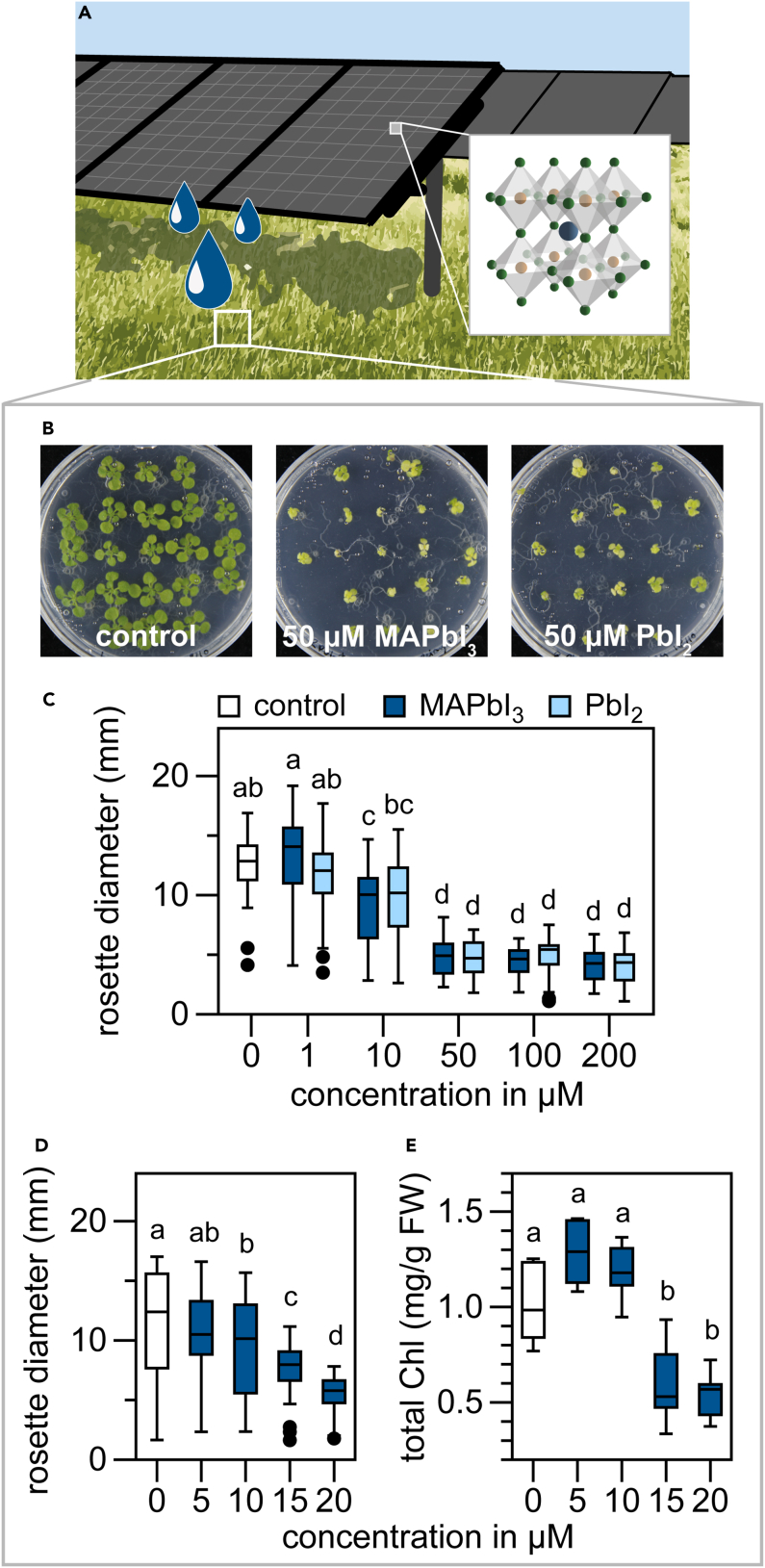
Lead halide perovskites inhibit *Arabidopsis* growth and chlorophyll accumulation (A) Schematic representation of agrivoltaics. The inset shows the structure of a lead halide perovskite. (B) Representative pictures of 18 day-old *Arabidopsis* plants, grown on control growth medium, or supplemented with methylammonium lead iodide (MAPbI_3_) or precursor PbI_2_. (C) Maximum rosette (plant) diameter in mm of 18 day-old *Arabidopsis* plants grown on growth medium supplemented with various concentrations of MAPbI_3_ or PbI_2_. (D) Maximum rosette diameter in mm and (E) total chlorophyll (ChlA + ChlB) content in mg/g fresh weight (FW) of 18 day-old *Arabidopsis* plants treated with various concentrations up to 20 μM of MAPbI_3_. For (C – E) Boxes represent the median +/− 25%, bars +/− 50%, black dots are outliers (n = 42). Different letters indicate statistical differences, with p < 0.05, tested by 1-way ANOVA and post-hoc Tukey’s test.

Sunlight provides enough energy to fulfill the global demand, making solar cells the most promising route toward a sustainable economy. Combining solar panels with agriculture, named *agri(photo)voltaics*, maximizes land use, while making optimum use of the sunlight for both crops and power generation (Figure 1A).(Adeh et al., 2019) Solar cells based on both silicon and lead halide perovskites (LHPs) are considered the next-generation commercial solar cells, with potential efficiencies up to >45%(Futscher and Ehrler, 2016; Leijtens et al., 2018). However, placing such next-generation solar cells on *e*.*g*., agricultural lands raises questions about the safety of LHPs for the environment. That is, if unintentional leakage releases Pb^2+^ ions in the soil, this may be harmful either to plants themselves or to humans and livestock through consumption of contaminated crops (Pourrut et al., 2011). A few studies have specifically tested the toxicity of the LHP MAPbI_3_ (MA = CH_3_NH_3_) and its supposedly less toxic tin-based counterpart on both plants and animals(Hailegnaw et al., 2015; Babayigit et al., 2016a, 2016b; Li et al., 2020). These studies focused on the heavy metals, but the presence of halides also raises environmental concerns (Medrano-Macías et al., 2016; Incrocci et al., 2019), which has not been studied in plants to date.

In this work, we find that the iodide in MAPbI_3_ causes greater harm to plants than the lead. Model species *Arabidopsis thaliana* was used to study the effect of MAPbI_3_ and different perovskite precursors on the growth and development of plants. Unlike previous work on perovskite toxicity (Babayigit et al., 2016a; Li et al., 2020), we avoid acidification effects by buffering the growth media to maintain a fixed pH, so we could selectively study the effect of the perovskite and its precursors. We used controlled growth settings with a high-resolution range of salt concentrations to define the exact toxicity thresholds. We find that the presence of MAPbI_3_ in the growth medium starts to affect plant performance at 5 μM and becomes significant at 10 μM. This concentration (10 μM) exceeds, according to our calculations, for a practical situation where one perovskite solar cell leaks into a similar soil area of 25 cm deep. In contrast to previous works, we conclude from experiments comparing the precursors MAI and PbI_2_ to MABr and PbBr_2_, that the iodide is responsible for inhibiting *Arabidopsis* development, before toxicity effects of lead appear. These results show that a more rigorous assessment on the potential harmfulness of LHPs is needed and stress the importance of developing strategies to avoid halides from being released into the environment.

## Results and discussion

Perovskites are fabricated from a lead halide salt, such as PbI_2_, and an organic halide salt, such as MAI (Figure 1A):

MAI + PbI_2_ → MAPbI_3_

Vice versa, decomposition of MAPbI_3_ leads to the formation of its precursors PbI_2_ and MAI. To assess the toxicity of LHPs, we germinated seeds of *Arabidopsis thaliana* (ecotype Columbia-0) on media containing different concentrations of MAPbI_3_, and several precursor salts. The media were buffered at a pH of 5.8, to avoid acidification effects (Babayigit et al., 2016a). Although seed germination is not affected, both MAPbI_3_ and PbI_2_ significantly inhibit plant growth at the seedling stage (depicted as rosette diameter) at concentrations >10 μM (Figures 1B and 1C). Growth inhibition stagnated from 50 μM, suggesting that no additional toxicity occurs beyond this concentration. To specify the level of lead perovskite toxicity, we grew plants at a range of MAPbI_3_ concentrations around 10 μM. As shown in Figure 1D, growth is significantly inhibited by concentrations >5 μM. In addition, plants appeared bleached when treated with higher concentrations of MAPbI_3_ or PbI_2_ (Figure 1B), which is supported by reduced chlorophyll levels in plants treated with over 10 μM of MAPbI_3_ (Figure 1E).

**Figure 2:**
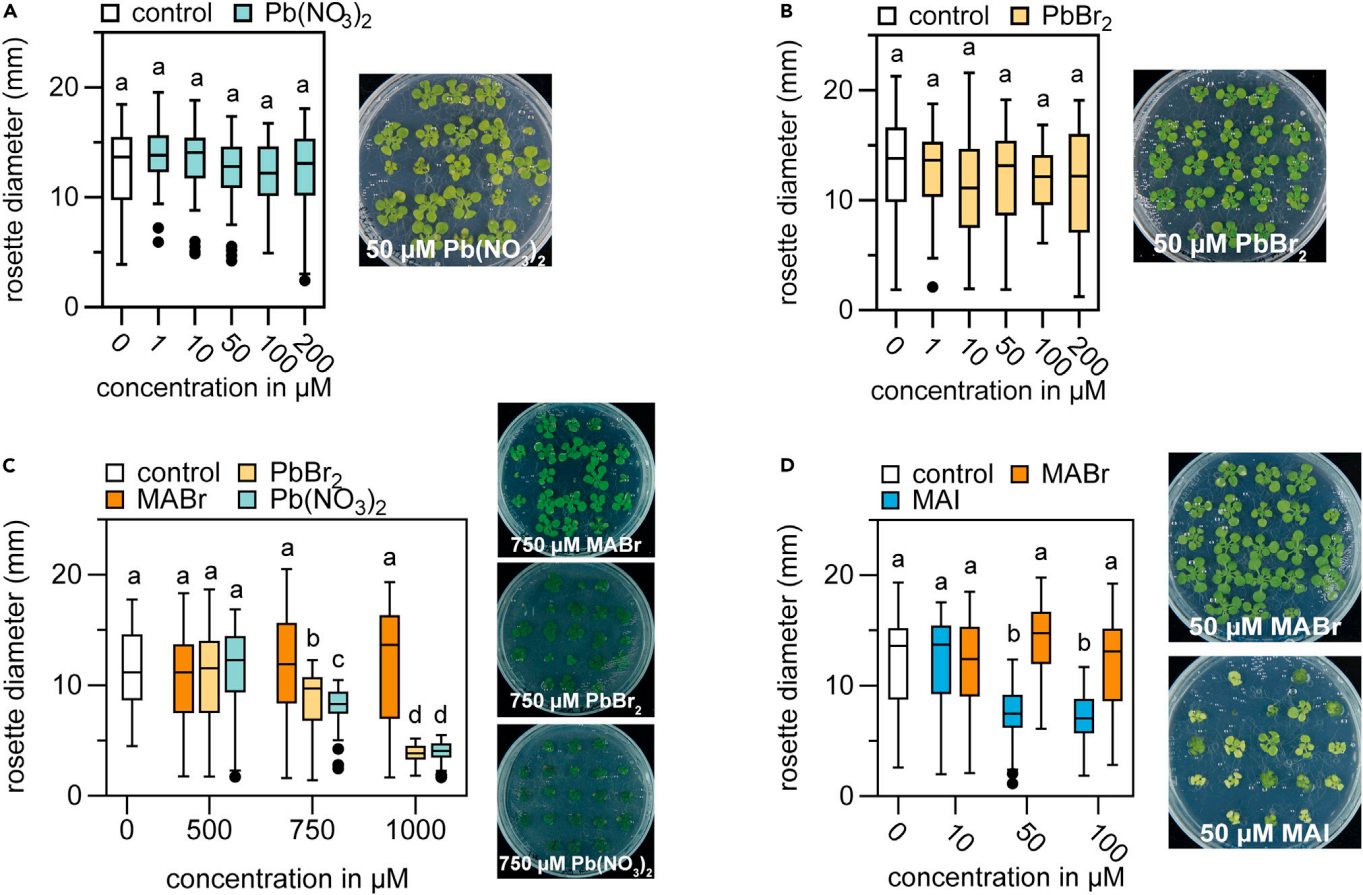
Iodide inhibits *Arabidopsis* growth at much lower concentrations than lead Maximum rosette (plant) diameter in mm of 18 day-old *Arabidopsis* plants grown on control growth medium, or supplemented with various concentrations of (A) Pb(NO_3_)_2,_ (B) PbBr_2_, (C) MABr, Pb(NO_3_)_2,_ and PbBr_2_, and (D) MABr and MAI. In (A – D), pictures show representative plants. Boxes represent the median +/− 25%, bars +/− 50%, black dots are outliers (n = 42). Different letters indicate statistical differences, with p < 0.05, tested by 1-way ANOVA and post-hoc Tukey’s test.

The Pb^2+^ oxidation state is toxic to plants (Pourrut et al., 2011; Babayigit et al., 2016b; Gupta et al., 2020). However, at concentrations for which we observed toxicity for both MAPbI_3_ and PbI_2_ (Figure 1C), lead nitrate (Pb(NO_3_)_2_) and another lead halide precursor (PbBr_2_) did not affect plant growth (Figures 2A and 2B). We found these lead-containing salts to significantly impede *Arabidopsis* growth at concentrations >750 μM (Figure 2C). This effect could be directly attributed to the lead, as MABr did not affect plant growth at these concentrations. Interestingly, even though growth was inhibited at high concentrations, Pb(NO_3_)_2_ and PbBr_2_ did not cause plant bleaching similar to MAPbI_3_ and PbI_2_ (compare Figures 1B and 2C). The observation that MAPbI_3_ and PbI_2_ hamper *Arabidopsis* growth at one order of magnitude lower concentrations (Figure 1) suggests that the iodide is at least in part responsible for this hampered growth. We repeated the growth experiments using the iodide precursor MAI and compared this to its bromide equivalent MABr (Figure 2D). As before, we found a significant inhibition of rosette size at 50 μM of MAI. In contrast, *Arabidopsis* growth is not affected by any concentration of MABr up to 1000 μM (Figures 2C and 2D).

Toxicity of MAPbI_3_ starts at 10 μM, and around 50 μM for MAI (Figure 2D), which we attribute to the three-fold higher concentration of available iodide ions in the MAPbI_3_ treatment. The hypothesis that the iodide is responsible for the toxicity at low MAPbI_3_ concentrations is further confirmed by the absence of toxicity of MABr and PbBr_2_ in the same concentration ranges (Figure 2C).

**Figure 3:**
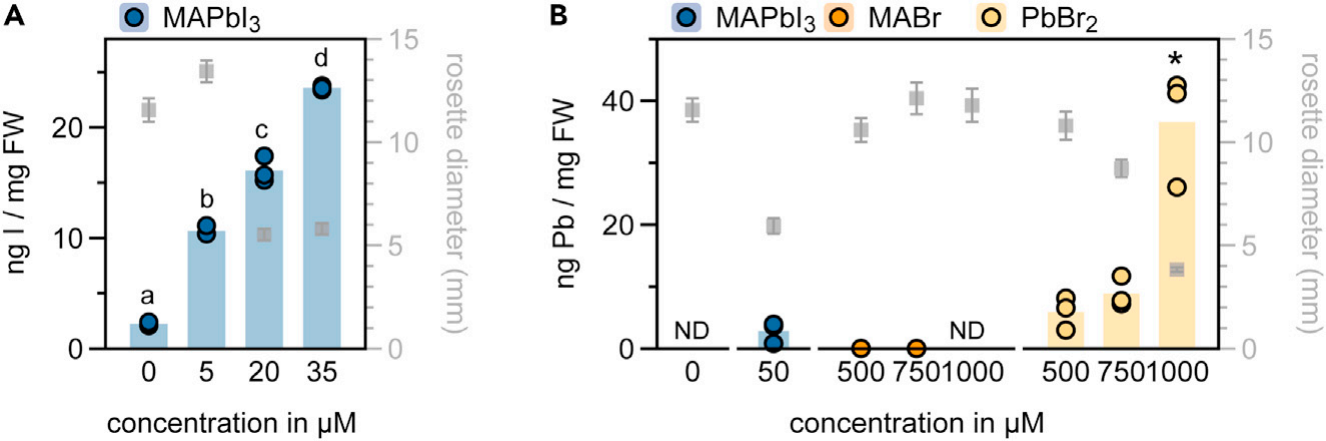
Growth inhibition caused by lead-iodide perovskites correlates strongly with iodine accumulation in *Arabidopsis* Concentrations of (A) iodine (I) and (B) lead (Pb) in ng/mg fresh weight (FW) in *Arabidopsis* plants grown on control growth medium (concentration = 0 μM), or supplemented with different concentrations of MAPbI_3_, MABr, and PbBr_2_. Bars represent averages of three biological replicates. Individual replicates are plotted as dots (n = 3). Different letters indicate significant differences. The asterisk in B indicates that this treatment was the only significantly different treatment from others. p < 0.05, tested by 1-way ANOVA and post-hoc Tukey’s test. ND = not detectable. Gray squares represent the average maximum rosette diameter in mm ± SE of 18-day old plants grown in the same treatments (n = 42). For control (in B), MABr and PbBr_2_ these are the same data as shown in Figure 2C.

Even though iodide is considered a (micro-) nutrient (Kiferle et al., 2021), the toxicity effects of iodide at higher concentrations are poorly understood. To confirm that iodide ions build up in the plants to reach a toxic level, we measured iodine content in *Arabidopsis* plants grown on different concentrations of MAPbI_3_ (Figure 3A). Iodine levels increased significantly in plants with increasing MAPbI_3_ concentrations. When comparing this to rosette size, we can conclude that in-plant levels >15 ng iodine/mg fresh weight caused growth inhibition in *Arabidopsis*. Similar to what was shown before, low concentrations of iodide correlated with slightly induced *Arabidopsis* growth (although not significant, see 1 μM in Figures 1C and 1D), most probably because of its nutritional value (Kiferle et al., 2021). This trend for iodide-mediated mild growth stimulation at low concentrations and toxicity at higher concentrations is not an *Arabidopsis*-specific phenotype, as it was seen before in strawberry, tomato, and various vegetable crops (Hong et al., 2009; Landini et al., 2011; Li et al., 2017).

Next, we measured Pb levels in *Arabidopsis* plants grown on 50 μM MAPbI_3_, or high (>500 μM) levels of MABr and PbBr_2_, see Figure 3B. As expected, Pb levels in control and MABr treated plants were extremely low or were not detectable. Plants treated with 50 μM MAPbI_3_ showed some accumulation, although not significant. The rosette size data shows that growth is strongly impaired in these plants when compared to the control treatment. However, similar Pb levels (approx. 10 ng Pb/mg fresh weight) were detected in plants treated with 500 or 750 μM PbBr_2_, but do not inhibit rosette size as severely in those cases. Only after applying 1000 μM PbBr_2_, plant growth is strongly repressed. These data support that increasing iodine levels in *Arabidopsis* plants treated with the LHP MAPbI_3_ are the main cause of toxicity and growth inhibition over increasing Pb levels. Our conclusion that the low toxicity threshold of MAPbI_3_ is caused by the presence of iodide, rather than lead, stresses the importance of further investigating the environmental effects of using iodide salts in solar panels.

Likely, the extent of toxicity of these halide salts in the environment will depend on the plant species, soil type, and depth to which the salts penetrate the soil in case of leakage. We estimate that a solar panel with a 400 nm thick perovskite layer contains 0.26 μmol of Pb^2+^ and 0.79 μmol of iodide (I^−^) per cm^2^. If the full panel would leak and be homogeneously distributed over a water column of the same area and 10 cm deep, this would yield concentrations of 26 μM (<5 mgkg^−1^ soil) Pb^2+^ and 79 μM iodide. Hence, the expected maximum concentration of lead in case of leakage is much lower than the concentration (>750 μM) at which it is toxic to the plant, and far below quality standards that range from 100 to 530 mgkg^−1^ depending on the country(Li et al., 2020). The concentration of iodide (79 μM), on the other hand, exceeds the toxicity threshold of 50 μM, so that the release of iodide into the environment upon leakage of a perovskite solar panel may affect plant growth and fitness.

To conclude, although the perovskite community so far has mainly discussed the potential toxicity of lead (Hailegnaw et al., 2015; Babayigit et al., 2016b; Slavney et al., 2016; Li et al., 2020), we found that iodide is toxic to *Arabidopsis* plants at one order of magnitude lower concentrations. We further find that lead is only significantly toxic at levels far above those expected for a solar module failure. Our observations stress the importance of getting a more complete picture on the potential harmfulness of solar panels that contain LHPs, especially if these are placed on agricultural lands, and to develop strategies to prevent the release of halides into the environment. The latter should primarily be realized by careful encapsulation of the solar cells to prevent leakage, or in case of unforeseen calamities, by considering halide-tolerant plants that accumulate leaked halides in a phytoremediation approach to clean the soil (Incrocci et al., 2019).

### Limitations of the study

In this study we have grown *Arabidopsis* seedlings *in vitro*, to be able to precisely apply treatments with perovskite salts. This helped to find a clear threshold for toxicity, especially iodide. In addition, we used a buffered solution to exclude effects from perovskite-induced changes in pH. However, in natural environments, where perovskites might leak from solar panels into the soil, toxicity of these compounds not only depends on their concentrations, but also on the to soil type, presence of microbiome, and other environmental factors such as water availability, pH, and the plant species. Additional experiments in natural settings would be required to test perovskite toxicity levels in natural conditions and for different plant species.

## STAR★Methods

### Key resources table

**Table:** 

REAGENT or RESOURCE	SOURCE	IDENTIFIER
**Chemicals, peptides, and recombinant proteins**		

Commercial bleach	N/A	N/A
SDS	VWR life science	Cat#0227
Murashige & Skoog inc. vitamins	Duchefa Biochemie	Cat#M0222.0050
Daishin agar	Duchefa Biochemie	Cat#D1004.1000
Potassium hydroxide (KOH)	Duchefa Biochemie	Cat#P0517.1000
Dimethyl sulfoxide (DMSO)	Sigma-Aldrich (now Merck)	Cat#41640
Lead iodide (PbI_2_)	TCI chemicals	Cat#L0279
Methylammonium iodide (MAI, methylamine hydroiodide)	TCI chemicals	Cat#M2556
Methylammonium bromide (MABr, methylamine hydrobromide)	TCI chemicals	Cat#M2589
Lead bromide (PbBr_2_)	Sigma-Aldrich (now Merck)	Cat#915696
Lead nitrate (Pb(NO_3_)_2_)	Sigma-Aldrich (now Merck)	Cat#467790

**Experimental models: Organisms/strains**		

*Arabidopsis thaliana* ecotype Col-0	N/A	N/A

**Software and algorithms**		

Fiji programme	Schindelin et al. 2012	https://imagej.net/software/fiji/
MVApp	Julkowska et al. 2019	https://mvapp.kaust.edu.sa

### Resource availability

#### Lead contact

Further information and requests for resources and reagents should be directed to and will be fulfilled by the lead contact, Charlotte M. M. Gommers (charlotte.gommers@wur.nl).

#### Materials availability

This study did not generate new unique reagents.

### Method details

#### Plant material and growth conditions

*Arabidopsis thaliana* ecotype Columbia-0 was used for all experiments. Seeds were surface-sterilized (20% bleach, 0.5%SDS), rinsed with sterile water and sown on half-strength Murashige & Skoog medium including vitamins (Duchefa Biochemie), containing 0,1% MES monohydrate buffer (Duchefa Biochemie) and 1% v/w Daishin agar (Duchefa Biochemie). Salts were added from a 1 mM stock in the desired concentration before autoclaving. The pH of all media was set at 5.8 using 0.1 N KOH to prevent any harmful acidification effects as described by Babayigit et al. (Babayigit et al., 2016a) Seeds were sown on petri dishes (9 cm diameter) containing 20 mL medium, stratified (4°C, dark) for 4 days to synchronize germination, and afterwards placed in a climate chamber (16 hours light period, 22°C, photosynthetic active radiation 150 μmol photons/m^2/^s).

#### Perovskite solutions

MAPbI3 was prepared by mixing and grinding the dry precursor powders in a stoichiometric ratio (0.4 millimole of MAI and 0.4 millimole of PbI2), using a mortar and pestle until a black powder was obtained. This was one under airtight conditions, inside a glovebox. Consequently, deionized water was added to the powder. On the addition of water, the powder immediately turned yellow, indicating decomposition of the perovskite into MAI (high solubility in water) and PbI_2_ (yellow, poor solubility in water). In addition, the separate precursors PbI_2_, PbBr_2_, MAI and MABr were dissolved in water at concentrations of 1 mM, as well as Pb(NO_3_)_2_ for reference experiments. The solutions for the experiments were prepared by dilution of the above stock solution. Concentrations of all salts used in this study, expressed in μM and g L^-1^ are given in Table S1.

#### Growth assays and chlorophyll quantification

Pictures of the plants were taken after 18 days of growth (2 petri dishes per treatment, 21 plants per petri dish). Maximum rosette diameter of all plants was analyzed using FIJI software (Schindelin et al., 2012). From each petri dish, three plants were harvested, weighed, and used for chlorophyll quantification: plants were shaken in 1 mL DMSO at 65°C in darkness for 1 hour, cooled and kept at room temperature for 30 minutes. Absorption was measured at 664 and 647 nm using a SpectraMax® Plus 384 Microplate Reader (Molecular Devices). Concentration of chlorophyll A and B was calculated by: [ChlA] (mg/L) = 12.25∗(A664) – 2.55∗(A647); [ChlB] (mg/L) = 20.31∗(A647) – 4.91∗(A664) (Porra et al., 1989). Values were converted into mg per mg fresh weight.

#### Element analysis

18-day old plants were harvested, pooled (2 plants per sample) in 50ml PE tubes and kept at -80°C until sent for analysis. Iodine (I) and lead (Pb) quantification by ICP-MS was performed by UT2A (Ultra Traces Analyses Aquitaine) at Pau University in France.

#### Estimated concentrations in the soil

Assuming a density of 4.09 gcm^-3^ for MAPbI_3_, (Li et al., 2020) and a thickness of 400 nm (4x10^-5^ cm), a solar panel contains 1.6x10^-4^ g perovskite per cm^2^. With molar weight fractions of 0.05 for MA^+^, 0.33 for Pb^2+^ and 0.61 for I^-^, this translates to 8.5x10^-6^ g MA^+^, 5.47x10^-5^ g Pb^2+^ and 1 x 10^-4^ g I^-^ per cm^2^ solar panel. If a full solar panel would break and leak into an equivalent area, the concentration in the soil will depend on the depth of leakage. For example, homogeneous distribution of the perovskite over the first 10 cm soil leads to concentrations of 8.5x10^-7^ g/cm^3^ MA^+^, 5.47x10^-6^ g/cm^3^ Pb^2+^ and 1 x 10^-5^ g/cm^3^ I^-^. Dividing these numbers by the respective molar weights, and considering that 1 cm^3^ = 1 mL, yields molarities of 26 μM MA^+^, 26 μM Pb^2+^ and 79 μM I^-^. If the soil has a density between 1 and 2 g/cm^3^, the concentrations are between 5 and 2.7 mgkg^-1^ for Pb^2+^ and between 10 and 5 mgkg^-1^ for the I^-^.

#### Statistical analysis

Statistical analyses were performed using the MVApp. (Julkowska et al., 2019). Levene’s test was used to test for equal variances. Multivariate comparisons were made with ANOVA, followed by a post-hoc Tukey test.

## Data Availability

• Data: The data reported in this paper will be shared by the lead contact upon request. • Code: This paper does not report original code. • Any additional information required to reanalyze the data reported in this paper is available from the lead contact upon request.
