# Supplementary material for: Metal halide perovskite toxicity effects on Arabidopsis thaliana plants are caused by iodide ions
Source: iScience. 2021 Dec 9;25(1):103583. doi: 10.1016/j.isci.2021.103583 (PMC8717450; doi:10.1016/j.isci.2021.103583)
Supplement: Document S1. Table S1 [file mmc1.pdf]

iScience, Volume *25*

Supplemental information

Metal halide perovskite toxicity effects on *Arabidopsis thaliana* plants are caused by iodide ions

Eline M. Hutter, Reiny Sangster, Christa Testerink, Bruno Ehrler, and Charlotte M.M. Gommers

Table S1. Concentrations of the different treatments used in this study, expressed in µM and in g L^{-1}. Related to the STAR Methods section.

**$MAPbI_3$** (MW: 619.966 g mol^{-1})	
conc. in µM	conc. in g L^{-1}
1	0.0006
5	0.0031
10	0.0062
15	0.0093
20	0.0124
35	0.0217
50	0.031
100	0.062
200	0.124

**MAI** (MW: 158.966 g mol^{-1})	
conc. in µM	conc. in g L^{-1}
10	0.0016
50	0.0079
100	0.0159

**$PbBr_2$** (MW: 367.008 g mol^{-1})	
conc. in µM	conc. in g L^{-1}
1	0.0004
10	0.0037
50	0.0184
100	0.0367
200	0.0734
500	0.1835
750	0.2753
1000	0.367

**MABr** (MW: 111.97 g mol^{-1})	
conc. in µM	conc. in g L^{-1}
10	0.0011
50	0.0056
100	0.0112
500	0.056
750	0.084
1000	0.112

**MABr** (MW: 111.97 g mol^{-1})	
conc. in µM	conc. in g L^{-1}
10	0.0011
50	0.0056
100	0.0112
500	0.056
750	0.084
1000	0.112

**$Pb(NO_3)_2$** (MW: 331.2098 g mol^{-1})	
conc. in µM	conc. in g L^{-1}
1	0.0003
10	0.0033
50	0.0166
100	0.0331
200	0.0662
500	0.1656
750	0.2484
1000	0.3312
